# Efficacy of intravenous methylene blue, intravenous artesunate, and their combination in preclinical models of malaria

**DOI:** 10.1186/1475-2875-13-415

**Published:** 2014-10-21

**Authors:** Colin Ohrt, Qigui Li, Nicanor Obaldia, Rawiwan Im-erbsin, Lisa Xie, Jonathan Berman

**Affiliations:** Walter Reed Army Institute of Research (WRAIR), Silver Spring, MD USA; Center for the Evaluation of Antimalarial Drugs and Vaccines, Tropical Medicine Research/Gorgas Memorial Institute, Panama City, Panama; Armed Forces Research Institute of Medical Sciences (AFRIMS), Bangkok, Thailand

**Keywords:** Methylene blue, Artesunate, Combinations, Malaria, Rat, *Aotus*, Rhesus

## Abstract

**Background:**

Intravenous artesunate (IV AS) is the present treatment of choice for severe malaria, but development of artemisinin resistance indicates that a further agent will be needed. Methylene blue (MB) is an approved human agent for IV and oral use, and is already being investigated for oral treatment of uncomplicated malaria. To initiate investigation of IV MB for severe malaria, the efficacy of IV MB was compared to IV AS and to their combination in rat and non-human primate malaria models.

**Methods:**

IV MB was compared to IV AS and to their combination in the *Plasmodium berghei*-infected rat, a self-curing model; the *Plasmodium falciparum*-infected *Aotus* monkey, a fatal model; and the *Plasmodium cynomolgi*-infected rhesus monkey, a fatal model. Key endpoints were clearance of all parasites from the blood and cure (clearance without recrudescence).

**Results:**

In rats, the minimal dose of individual drugs and their combination that cleared parasites from all animals was 20 mg IV MB/kg/day, 60 mg IV AS/kg/day and 10 mg IV MB/kg/day plus 30 mg IV AS/kg/day. In *Aotus*, 8 mg IV MB/kg/day and 8 mg IV AS/kg/day each cured two of three monkeys by one day after therapy, and the third monkey in each group was cured two days later. The combination of both drugs did not result in superior efficacy. In rhesus, 8 mg IV MB/kg/day and 8 mg IV AS/kg/day performed comparably: parasite clearance occurred by day 3 of therapy, although only one of four animals in each dose group cured. Eight mg/kg/day of both drugs in combination was 100% successful: all four of four animals cured.

**Conclusions:**

In each of the three animal models, the efficacy of IV MB was approximately equal to that of standard of care IV AS. In the rat and rhesus models, the combination was more effective than either single agent. This preclinical data suggests that IV MB, alone or in combination with IV AS, is effective against *Plasmodium spp.* and can be evaluated in severe malaria models.

## Background

Severe malaria is a medical emergency and requires parenteral, preferably intravenous (IV), treatment. Even with the classic standard of care IV quinine, mortality is approximately 10 to 20% and generally occurs within two to five days [1, 2]. The new treatment of choice is IV artesunate (AS), which reduced mortality compared with quinine in two large Phase III trials [1, 2]. Although initial IV treatment for approximately three days kills the vast majority of parasites, these short-half-life drugs do not eliminate all parasites from the body. Once the patient is able to receive oral treatment, cure (elimination of all parasites to prevent recrudescence) is accomplished by administration of longer half-life oral agents.

Oral artemisinin combinations are now the standard of care worldwide for uncomplicated malaria, and early reports of clinical resistance to AS are emerging from Cambodia [3, 4]. The development of resistance to artemisinins indicates that another agent in addition to IV AS and IV quinine will be needed for initial treatment of severe malaria.

IV Methylene blue (MB) is approved worldwide to treat methaemoglobinaemia. MB has been investigated in vitro and as an oral anti-malarial agent in the clinic. The biochemical mechanism of MB’s approved use is to reduce oxidized haemoglobin (methaemoglobin) back to haemoglobin, and it is hypothesized that MB kills *Plasmodium spp* via an increase in oxidants [5]. In vitro, MB is active against both chloroquine-sensitive and chloroquine-resistant parasites [6]. Oral MB was tested for treatment of uncomplicated malaria in a region of chloroquine resistance in Burkina Faso (37% early treatment failures with chloroquine). Although MB at a dose of 4 mg/kg/day for three days in combination with chloroquine was ineffective (29% early treatment failures [7]), a higher dose of MB, 12 mg/kg twice a day for three days, with chloroquine was effective: there were no early treatment failures [8]. A dose of MB at 10 mg/kg twice a day for three days was then evaluated in combination with AS or with amodiaquine. Both MB-plus-AS and MB-plus-amodiaquine were effective initially: there were no early treatment failures in either group. Recrudescence was seen after MB plus AS but not after MB plus amodiaquine [9]. These data are reasonable given that the half-life of MB in humans is 15–19 hours [10], which means that MB needs to be partnered with a long half-life drug such as amodiaquine to prevent recrudescences.

The focus of clinical work to date with MB is on the oral treatment of uncomplicated malaria. To initiate the investigation of IV MB for severe malaria, the efficacy of IV MB was compared to IV AS and to their combination in rat and non-human primate models of malaria.

## Methods

### Rat experiments

#### Drugs and chemicals

Artesunic acid (AS: Knoll AG, BASF Pharmaceuticals) was formulated as an AS-lysine salt by dissolving AS in 5% of a DL-2,6-diaminohexanoic acid (DL-lysine) solution. MB salt was purchased from Aldrich Chemical Co (Milwaukee, WI, USA). L-lysine (monohydrochloride), which was used as control agent, and DL-lysine were obtained from Sigma Chemical Co (Pittsburgh, PA, USA).

### Animals and parasites

Groups of six to ten seven-week old Spraque-Dawley rats were inoculated with a rat-adapted strain of *Plasmodium berghei* ANKA as described previously [11]. *Plasmodium berghei* is a self-curing parasite in rats: parasitaemia spontaneously diminishes beginning approximately day 12 after inoculation.

### Study design

First, each drug (IV AS or IV MB) was administered singly using a sequential dose-escalation design. Each successive group of rats was administered a higher dose of drug until the maximum tolerated dose (MTD) was achieved. The MTD was defined as the highest dose that did not cause death in any animals. Then, combinations of MB and AS were administered again until the MTD was achieved. In the combination experiments, IV AS was injected first followed immediately by MB.

### Study procedures

On study day 1, rats were inoculated intraperitoneally with cryopreserved *P. berghei*-infected rat blood. When parasitaemia was >4% on day 6, treatment was administered daily for three days. Post-treatment, parasitaemia was evaluated via tail-blood sampling at zero, three, five, eight, and 12 hours on day 6; zero, three and six hours on day 7 and 8; and daily from day 9 to day 12. The experiments were performed in 2004.

### Efficacy parameters and analysis

#### Determination of parasitaemia

Thin smears were examined under oil at 1,000× magnification. Parasitaemia was calculated using the formula: parasitaemia (%) = [number of infected red blood cells/(number of infected red blood cells + number of non-infected red blood cells)] × 100. A negative smear was defined as the failure to observe a parasite after examination of 10,000 red blood cells (i.e., <0.01% parasitaemia).

Parasite clearance was defined as a negative blood smear by day 12. Day 12 was taken as the end of the time period during which clearance could be demonstrated since in this model, the animals begin to self-cure their infection on approximately this day [12]. The parasite clearance time was defined as the time between the beginning of dosing and the first negative blood smear.

The therapeutic index was defined as minimal toxic dose/minimal clearance dose.

### *Aotus*experiments

#### Drugs and chemicals

AS was purchased from Guilin #2 Pharmaceutical Factory, Guangxi, China. MB salt (3,7-Bis (dimethylamino) phenthiazin-5-ium chloride) was purchased from Aldrich Chemical Co (Milwaukee, WI, USA) and dissolved in 0.9% sodium chloride.

### Animals and parasites

Data were obtained from male and female laboratory-bred *Aotus lemurinus lemurinus* monkeys Karyotype VIII and IX [13], weight 692–1,345 g. The animals were housed at Gorgas Memorial Institute of Health Studies (ICGES) in Panama, and cared and maintained as described elsewhere [14].

Experimental monkeys were challenged with parasites using methods patterned after Schmidt [15]. Each parasite inoculum contained 5 × 10^6^*Plasmodium falciparum-*parasitized erythrocytes (FVO strain) in a volume of 1.0 mL. Non-splenectomized monkeys were used in these experiments. *Plasmodium falciparum* (FVO strain) is generally fatal in *Aotus*. In model development, of 293 monkeys that were infected and untreated, 149 (50%) died within 15 days, 84 (29%) died within 30 days, and only 33 (11%) self-cured [15].

### Study design

For the first study, infected monkeys were randomly assigned to four dosage groups: MB 0 mg/kg/day × three days (MB0 - non drug treated control), MB 8 mg/kg/day × three days (MB8), MB 16 mg/kg/day × three days (MB16), MB 24 mg/kg/day × three days (MB24). There was one monkey in the MB0 group and two monkeys in each of the three experimental groups.

For the second study, infected monkeys were randomly assigned to five dosage groups: MB0, MB 4 mg/kg/day × three days plus AS 4 mg/kg/day × three days (MB4 plus AS4), MB 8 mg/kg/day × three days (MB8), AS 8 mg/kg/day × three days (AS8), MB 8 mg/kg/day × three days plus AS 8 mg/kg/day × three days (MB8 plus AS8). There were two monkeys in the MB0 group and three monkeys in each of the four experimental groups.

### Study procedures

Blood smears were performed daily beginning four days after parasite inoculation. Treatment was initiated when the cohort’s parasitaemia level reached an aggregate mean treatment threshold of approximately 50,000/mm^3^ [1%]. Treatment was given as a single IV injection of the designated drug or immediate sequential injection of both drugs in a combination once daily for three consecutive days. The experiments were performed in 2005.

### Endpoints

Parasite counts were done by the Earle and Perez method [16], using daily Giemsa-stained thick blood smears obtained from a prick in the marginal ear vein.

Response to treatment was categorized as no effect, suppression without clearance, clearance and recrudescence, or clearance and cure. The day of clearance was defined as the first of three consecutive days in which the thick blood films were parasite negative. Suppression was defined as a transient decrease in parasite density post-treatment without clearance [17]. Animals that cleared parasitaemia were followed for recrudescence with daily blood sampling for parasite determinations until the end of the experiment on day 24 (first experiment) or daily blood sampling until day 32 then biweekly blood sampling until the end of the experiment on day 105 (second experiment). The day of recrudescence was the first of three consecutive days of positive thick blood films after a period of clearance. Animals that cleared and did not recrudesce by the end of the experiment were considered cures.

### Rhesus experiments

#### Drugs and chemicals

AS was purchased from Guilin #2 Pharmaceutical Factory, Guangxi, China. For cohort 1, AS was dissolved as a 5% solution in sodium bicarbonate and administered without further dilution. In cohort 2, AS was dissolved as a 5% solution in sodium bicarbonate then diluted with five volumes of 5%-dextrose-in-water before being administered. MB salt (3,7-Bis (dimethylamino) phenthiazin-5-ium chloride) was purchased as a 1% solution (American Reagent Laboratories Inc, Shirley, NY, USA) and administered without dilution.

### Animals and parasites

Experimental monkeys were challenged with parasites using methods patterned after those described by Schmidt [18]. Each parasite inoculum contained 1 × 10^6^*Plasmodium cynomolgi bastianellii*-parasitized erythrocytes in a volume of 1.0 mL. Splenectomized *P. cynomolgi*-naïve rhesus monkeys were used in these experiments. *Plasmodium cynomolgi* is fatal to splenectomized rhesus if untreated. The experiments were performed in 2004.

### Study design

Animals were randomly assigned to nine groups: controls AS 0 mg/kg/day (AS0) plus MB 0 mg/kg/day (MB0) to which two animals were assigned, and eight experimental groups to which four animals were assigned. The experimental groups were: AS0 plus either MB 2 mg/kg/day (MB2) or MB 8 mg/kg/day (MB8); AS 1 mg/kg/day plus either MB0 (AS1), MB2 (AS1 plus MB2), or MB8 (AS1 plus MB8); AS 8 mg/kg/day plus either MB0 (AS8), MB2 (AS8 plus MB2), or MB8 (AS8 plus MB8). In this way, evaluations were made of either drug alone at low and high doses, and the combination of each drug at low and high doses with the low and high doses of the other drug. Each dose of drug was administered once a day intravenously for three consecutive days.

### Study procedures

There were two cohorts of 17 monkeys each. Each cohort consisted of one control monkey and two monkeys for each of the eight treatment groups. Blood smears were performed daily beginning on the day before the parasite inoculation until parasitaemia exceeding 5,000 per mm^3^ were measured. For cohort 1, treatment was initiated when the cohort’s parasitaemia level reached a mean of 8% on day 9 after parasite inoculation. Because the untreated control animal in cohort 1 died one day later, treatment for cohort 2 was initiated when mean parasitaemia reached 5% on day 8 after parasite inoculation. Treatment was given via a single injection of the designated drug or immediate sequential injection of the two drugs in a combination via a peripheral vein once daily for three consecutive days.

Once treatment was initiated, blood smears were performed every two hours for the first 12 hours after treatment, then every 12 hours at 24, 36, 48, 60, and 72 hours, then twice daily until parasitaemia cleared, then daily for four weeks and twice weekly for two weeks and once weekly for two more weeks.

### Endpoints

Parasites were enumerated as per the rat experiments. Per cent parasitaemia was calculated as per the rat experiments.

Clearance was defined as being without observable parasites for five consecutive days. The parasite clearance time was defined as the time of the first negative smear. Recrudescence was defined as the presence of patent parasitaemia after clearance. Cure was defined as clearance without recrudescence by the end of the experiment.

### Animal use

Protocols were approved by the WRAIR Institutional Animal care and Use Committees (IACUC) (rat experiments); Gorgas Memorial Institute IACUC (Aotus experiments), and AFRIMS IACUC (Rhesus experiments). Animals were maintained in accordance with established principles under the *Guide for the Care and Use of Laboratory Animals* [19].

## Results

### Rats infected with *Plasmodium berghei*

#### Control animals

Mean parasitaemia in this group increased to approximately 5% on day 6, at which point all but the control animals were treated. In control animals, parasitaemia rose to approximately 10% on day 8, reached a peak on day 11 between 10 and 35%, and then declined (self cleared) to undetectable levels over the next seven to 11 days in the surviving animals. A 16.7% mortality from malaria was observed in the control rats (Table 1).

**Table 1:** **Efficacy of intravenous methylene blue, intravenous artesunate, and their combination in the** ***Plasmodium berghei*** **-infected rat**

A) Methylene blue alone							
Dose (mg/kg/day × 3 days)	0	5	10	20	40	80	
Number of rats	6	7	7	7	8	7	
Number of rats cleared*	0/6	0/7	0/7	7/7	8/8		
PCT (mean hour)				52	48		
Mortality (dead/total)	1/6	1/7	0/7	0/7	0/8	7/7	
**B) Artesunate alone**							
Dose (mg/kg/day × 3 days)	0	15	30	60	120	240	480
Number of rats	6	7	6	6	6	6	5
Number of rats cleared*	0/6	0/7	0/6	6/6	6/6	6/6	
PCT (mean hour)				59	48	49	
Mortality (dead/total)	1/6	1/7	0/6	0/6	0/6	0/6	4/5
**C) Methylene blue in combination with artesunate**							
Dose (mg/kg/day × 3 days)	MB5/AS 15	MB10/AS30	MB20/AS60	MB40/AS120	MB40/AS240		
Number of rats	8	8	12	6	5		
Number of rats cleared*	4/8	8/8	12/12	6/6			
PCT (mean hour)	61	46	45	36			
Mortality (dead/total)	0/8	0/8	0/12	0/6	4/5		

*Clearance signifies no parasitaemia by day 12 after infection.

### Efficacy of intravenous methylene blue alone

No rats administered 5 or 10 mg/kg/day cleared by day 12. All rats administered 20 or 40 mg/kg/day cleared, with an average clearance time of approximately 50 hours. Since 80 mg/kg/day was toxic (mortal) to all rats, the therapeutic index was calculated to be 4.

### Efficacy of intravenous artesunate alone

No rats administered 15 or 30 mg/kg/day cleared their parasites. All rats administered 60, 120 or 240 mg/kg/day cleared their parasites with an average clearance time of approximately 50 hours. Since 480 mg/kg/day was toxic to all rats, the therapeutic index was calculated to be 8.

### Efficacy of intravenous methylene blue in combination with intravenous artesunate

Some rats cleared their parasites in each group that received combination therapy. Half of the rats in the MB 5 mg/kg/day plus AS 15 mg/kg/day cleared their parasites; all rats in the MB 10 mg/kg/day plus AS 30 mg/kg/day group, the MB 20 mg/kg/day plus AS 60 mg/kg/day group, and the MB 40 mg/kg/day plus AS 120 mg/kg/day group cleared their parasites. Clearance times were again close to 50 hours, except for the highest dose group. Since MB 40 mg/kg/day plus AS 480 mg/kg/day was toxic to all rats, the therapeutic index was calculated to be between 4 (for MB) and 8 (for AS).

### *Aotus*monkeys infected with *Plasmodium falciparum*

#### Control animals

Parasitaemia in all animals increased from approximately 2,000 parasites/mm^3^ on day 4 after infection to at least 50,000 parasites/mm^3^ on day 5 after infection, at which point all but the control animals were treated. By day 8, the three control monkeys had parasitaemia of 600,000-800,000 parasites/mm^3^ and were given rescue therapy.

### Experiment No 1: intravenous methylene blue alone

IV MB doses of 8, 16 and 24 mg/kg/day × three days each cleared 100% of parasites by the fifth day after the beginning of therapy, although no dose prevented recrudescence which occurred on days 7–9 (Table 2 top).

**Table 2:** ***Aotus*** **experiments**

Experiment No 1 methylene blue alone		
**IV MB dose (mg/kg/day × 3 days)**	Clearance day*	Recrudescence day*
8	5	7
8	5	7
16	5	7
16	5	9
24	5	7
24	5	8
**Experiment No 2 methylene blue plus artesunate**		
Drug regimen**	Clearance day*	Recrudescence day*
AS8	4	13
AS8	4	9
AS8	5	8
MB8	4	7
MB8	4	7
MB8	6	7
MB8 plus AS8	3	9
MB8 plus AS8	(Not clear)	
MB8 plus AS8	7	9

*Day 1 is the first day of treatment. Day 4 is the first day after three days of therapy.

**MB4, MB8, AS4, and AS8 signify IV MB 4 mg/kg/day × three days, MB 8 mg/kg/day × three days, IV AS 4 mg/kg/day × three days, and AS 8 mg/kg/day × three days, respectively.

Each row represents data from one animal.

### Experiment No 2: intravenous methylene blue plus intravenous artesunate

The purpose of this experiment was to compare malaria treatment with 8 mg/kg/day IV MB, the lowest dose tested in experiment No 1 which cleared 100% of parasites in that experiment, to treatment with 8 mg/kg/day of IV AS and to the combination of IV AS and IV MB (Table 2 bottom).

As single agents, both drugs cleared 100% of parasites in each of three animals, although neither drug prevented recrudescence on days 7–13. The mean values for clearance day were approximately equal, although MB delayed recrudescence longer than did AS. For the combination of MB8 plus AS8, one animal did not clear, and the days to clearance and recrudescence for the two other animals were not superior to the values for the animals given single agents.

Two animals in the AS8 group had haematological abnormalities that might be interpreted as due to continued parasitaemia. One animal had significant thrombocytopaenia (<50 × 10^3^ × uL) on day 44 post-therapy. Another animal had severe anaemia on day 50 post therapy. These changes in haematologic indices could reflect subpatent parasitaemia, but if so, parasitaemia did not become patent by the end of the experiment on day 105. Two animals in the MB8/AS8 group had significant loss of weight or decrease in platelets on days 51 and 56 post therapy, respectively, but survived to the end of the experiment on day 105 and were considered cured.

### Rhesus monkeys infected with *Plasmodium cynomolgi*

#### Control animals

The control animal administered AS0 plus MB0 died on day 1 of ‘therapy’ (cohort 1) or was given rescue therapy when parasitaemia reached 33% on day 2 of ‘therapy’ (cohort 2) (Table 3).

**Table 3:** **Rhesus experiment**

MB dose*	AS dose*	Clearance day**	Recrudescence day** or cure
0	0	Died day 1	Not applicable
0	0	Rescued day 2	Not applicable
2	0	Not clear	Not applicable
2	0	10	Recrudescence day 12
2	0	Not clear	Not applicable
2	0	Not clear	Not applicable
8	0	2	Cure
8	0	1	Recrudescence day 12
8	0	1	Recrudescence day 12
8	0	1	Recrudescence day 14
0	1	2	Recrudescence day 11
0	1	3	Recrudescence day 9
0	1	3	Recrudescence day 9
0	1	1	Recrudescence day 7
2	1	1	Cure
2	1	1	Cure
2	1	3	Recrudescence day 5
2	1	1	Recrudescence day 15
8	1	1	Cure
8	1	1	Recrudescence day 15
8	1	4	Cure
8	1	1	Cure
0	8	Died day 2	Not applicable
0	8	1	Cure
0	8	3	Recrudescence day 12
0	8	3	Recrudescence day 11
2	8	Died day 3	Not applicable
2	8	Died day 2	Not applicable
2	8	3	Cure
2	8	3	Cure
8	8	1	Cure
8	8	1	Cure
8	8	1	Cure
8	8	1	Cure

*mg/kg/day × three days.

**Day 1 is the first day of treatment. Day 4 is the first day after three days of therapy.

Each row represents data from one animal.

### Drug-treated animals

The efficacy of AS alone, MB alone, or their combination is shown in Table 3 for each animal. MB2 was relatively ineffective. Three animals never cleared and the day of parasite clearance of the animal that did clear was prolonged. MB8 was more effective than MB2, since all animals cleared by day 2 and one did not recrudesce. AS1 caused all animals to clear, although each animal later recrudesced. AS8 was more effective than AS1 in the sense that one of the AS8 animals cured, but one of the AS8 animals died on the first day of therapy of causes unknown.

For the combination groups, clearance times ranged from two to three days. Although two monkeys in the AS8 plus MB2 group died in the course of therapy, these deaths were unlikely due to drug toxicity, since there were no deaths in the MB8 plus AS8 group. MB8 plus AS1 therapy was very effective, with all four of four animals clearing their parasites by day 4 and three of four animals being cured. The MB8 plus AS8 group was strikingly effective, with all four of four animals clearing parasites by day 1 and all animals being cured.

## Discussion

Investigation of IV MB as a potential agent for severe malaria is driven by the likely impending need for an alternative to or supplementation of IV AS, as artemisinin resistance spreads beyond Southeast Asia.

IV MB has the advantage of already being approved worldwide as a treatment for methaemoglobinaemia. The recommended dose is 1–2 mg/kg repeated once if necessary, with 7 mg/kg being the ‘prescribing limit’ [20, 21]. Other present clinical uses of MB are as a dye to localize parathyroid tumours prior to surgery, for which a single dose up to 7.5 mg/kg has been used [22], and to treat ifosfamide-associated encephalopathy since MB inhibits monoamine oxidase conversion of ifosfamide to an encephalotoxic metabolite [23], for which approximately 1 mg/kg six times over one day was administered in one study [24].

Oral MB has already been investigated as a treatment for uncomplicated malaria. Since the bio-availability of MB is approximately 70% [10], the MB regimens of 10–12 mg/kg twice a day for three days that were used in combination with oral chloroquine, AS or amodiaquine, are pharmacokinetically equivalent to an intravenous regimen of 7 mg/kg twice daily for three days. Side effects in the oral MB plus AS group that were not present in the amodiaquine-plus-AS group were an increased frequency of vomiting and dysuria [9]. If the mechanism of vomiting in response to oral MB is local, treatment with IV MB would not cause vomiting. A further consideration for the use of IV MB for the severe malaria indication is that MB may modulate host physiology during shock. Guanylate cyclase is activated during septic shock to produce cyclic guanosine monophosphate, which in turn leads to relaxation of myocardial and vascular smooth muscle and an increase in vascular permeability. MB is an inhibitor of guanylate cyclase and thus could lead to vasoconstrictive and positive inotropic effects during shock states [25]. Another possible concern is the generation of methaemoglobin from haemoglobin, which, for example, equaled 5% when one patient was administered 7 mg/kg in a case report [26]. In this regard, severe malaria patients treated with artesunate sometimes experience a delayed hemolytic episode [27]. It is not likely that there would be pharmacokinetic interactions between AS and MB. Artesunate is converted in vivo to dihydroartemisinin [28], which is glucuronidated in the liver [29]. Methylene blue is rapidly reduced to leucomethylene blue in tissues [21].

Based on the hypothesis that IV MB at a dose of 7 mg/kg either daily or twice daily for three days might have utility for severe malaria as a replacement for, or in combination with, IV AS, IV MB was evaluated in rat and non-human primate malaria models. Dosing was daily for three days, the approximate length of time that intravenous drugs are administered in the initial treatment of severe malaria in humans.

In rats infected with *P. berghei*, the minimal dose of IV MB that cleared parasites from all animals was 20 mg/kg/day and the minimal dose of IV AS that cleared parasites from all animals was 60 mg/kg/day. Although this comparison suggests that MB is superior to AS, the 100% toxic doses of these drugs were 80 mg/kg/day (MB) *vs* 480 mg/kg/day (AS) and thus AS had a superior therapeutic index. The combination of MB plus AS was more effective than either drug alone, since MB 10 mg/kg/day plus AS 30 mg/kg/day cleared all parasites. The implication that MB plus an artemisinin is more effective in rodents than either drug alone is consistent with a recent brief report of a mouse model. In C57BL6/N mice infected with *P. berghei,* five days of intraperitoneal treatment with MB (10 mg/kg) or dihydroartemisinin (3 mg/kg) permitted survival only to 9 days or 25 days, respectively, but the combination permitted survival of 80% of animals through 45 days [30].

In *Aotus* infected with *P. falciparum,* IV MB at 24 mg/kg/day, 16 mg/kg/day and 8 mg/kg/day cleared parasites by two days after the end of therapy. In the subsequent *Aotus* experiment, IV MB at the lowest previously tested dose (8 mg/kg/day) was compared to IV AS at the same dose. For both MB and AS, all monkeys cleared by three days after the end of therapy, although each monkey recrudesced soon thereafter. The combination of both drugs at a dose of 8 mg/kg/day in this model did not result in efficacy data that were superior to either drug alone.

In rhesus infected with blood stages of *P. cynomolgi*, IV MB and IV AS were tested at a low dose (2 mg/kg/day for IV MB, 1 mg/kg/day for IV AS) and at a high dose of 8 mg/kg/day, alone and in combination. At the lower doses, AS was superior to MB in terms of clearance, since all of the AS animals cleared but three of the four MB-treated animals did not. At 8 mg/kg/day, IV MB and IV AS performed comparably: parasite clearance occurred by day 3 of therapy, except for one AS animal that died during treatment.

A high dose of MB (8 mg/kg/day) plus a low dose of AS (1 mg/kg/day) appeared more effective than either drug alone: three of four animals cured. A high dose of AS (8 mg/kg/day) plus a low dose of MB (2 mg/kg/day) also appeared effective (two of four animals cured) although two animals died during treatment for unknown reasons. The combination of high doses of MB and AS (8 mg/kg/day for each drug) was 100% successful: all four of four animals cured.

Overall, the efficacy of MB was approximately equal to that of standard of care AS in each of the three animal models. In two of the models, rat and rhesus, the combination was more effective than either agent alone. The comparable efficacy of IV MB to IV AS, and the high cure rate of the high dose of MB plus either the low dose of AS or the full dose of AS, in splenectomized rhesus is noteworthy given that this is a non-human primate model for which untreated infections are fatal.

These data suggest that IV MB and combinations of IV MB and IV AS have potential for malaria treatment. Further investigation might be undertaken in a non-human-primate model of cerebral malaria, the *Plasmodium coatneyi*-infected rhesus monkey [31]. Additionally, since both IV MB and IV AS are clinical agents, IV MB alone and in combination with IV AS might be evaluated in progressively more severe cases of clinical malaria.

## Conclusion

IV MB, an approved clinical agent, was approximately as effective as IV AS, the standard of care for severe malaria, in preclinical rat and non-human primate models. The combination of both drugs was more effective than either drug alone in the *P. cynomolgi-*infected rhesus monkey*,* for which untreated infections are fatal. This large preclinical experience suggests that IV MB, alone or in combination with IV AS, be evaluated in preclinical models of severe malaria and potentially in clinical severe malaria.

## Abbreviations

AS: Artesunate
IACUC: Institutional Animal Care and Use Committee
IV: Intravenous
MB: Methylene blue
MTD: Maximum tolerated dose.
